# New-Onset Post-Operative Atrial Fibrillation in Patients Undergoing Coronary Artery Bypass Grafting Surgery - A Retrospective Case-Control Study

**DOI:** 10.21470/1678-9741-2021-0220

**Published:** 2023

**Authors:** Xuehui Cui, Can Xu, Cheng Chen, Yunyan Su, Jie Li, Xiaojun He, Dongjin Wang

**Affiliations:** 1 Department of Thoracic and Cardiovascular Surgery, the Affiliated Drum Tower Hospital of Nanjing University Medical School, Nanjing, China.; 2 Department of Cardiology, the Affiliated Drum Tower Hospital of Nanjing University Medical School, Nanjing, China.; 3 Department of Cardiovascular Surgery, Ruijin Hospital, Shanghai Jiao Tong University School of Medicine, Shangai, China.

**Keywords:** Atrial Fibrilation, Blood Pressure, Coronary Artery Bypass, Echocardiography, Diuretics, Drainage, Hospital, Postoperative Complications, Risk Factor

## Abstract

**Introduction:**

New-onset postoperative atrial fibrillation (POAF) is a common complication following coronary artery bypass grafting (CABG) surgery.

**Objective:**

To explore predictive factors and potential mechanisms of new-onset POAF in isolated off-pump CABG patients.

**Methods:**

Retrospective observational case-control study of 233 patients undergoing isolated off-pump CABG surgery between August 2018 and July 2020 at the Department of Thoracic and Cardiovascular Surgery, the Affiliated Drum Tower Hospital of Nanjing University Medical School. Associations between predictor variables and new-onset POAF were identified. The main outcome was new-onset POAF after CABG surgery.

**Results:**

A total of 75 (32.19%) patients developed new-onset POAF after CABG surgery. The new-onset POAF patients had advanced age, higher baseline systolic blood pressure, more preoperative use of diuretic drug, more transfusion of blood products, atrial dilation and postoperative positive inotropic drug treatment. Nineteen variates entered the multivariable logistic regression model with a Hosmer-Lemeshow test score of 7.565 (P=0.477). Postoperative left atrial enlargement, postoperative drainage in the first 24 hours and total length of hospital stay were statistically significant, while postoperative right atrial enlargement (OR and 95% CI, 7.797 [0.200, 304.294], P=0.272) and left atrial enlargement (3.524 [1.141, 10.886], P=0.029) assessed by echocardiography had the largest OR value.

**Conclusion:**

Atrial enlargement is strongly associated with new-onset POAF in patients with isolated off-pump CABG, thus it highlights the advantage of echocardiography as a useful tool for predicting new-onset POAF. Careful monitoring and timely intervention should be considered for these patients.

## INTRODUCTION

New-onset postoperative atrial fibrillation (POAF) is a frequent complication of coronary artery bypass grafting (CABG) surgery, with a reported incidence between 11% and 50%^[1]^. It tends to occur within 2 to 4 days after the procedure, with a peak incidence on postoperative day 2^[2]^. Patients with POAF are at increased risk of perioperative complications, including thromboembolic events, stroke, prolonged hospital stay, in-hospital mortality and long-term mortality compared with patients who remain in sinus rhythm after CABG surgery^[3-5]^. Overall POAF rates have persisted over decades of attempted interventions^[6]^. The underlying mechanisms are not well established, and different risk factors such as advanced age, hypertension, heart failure, myocardial infarction, atrial fibrosis, atrial dilation and obesity have been reported^[7]^. Even though recent advancements indicate that POAF is in part preventable by anti-inflammatory therapy^[8]^, progress has been hindered by scarce and conflicting data, and lack of knowledge on independent predictors, effective interventions^[7]^. Therefore, we performed this retrospective case-control study to explore risk factors of the new-onset POAF, trying to add valuable data for future research and clinical decisions. Since most of our patients underwent off-pump operation for isolated CABG, we focus on this type of patient to reduce heterogeneity and potential bias. We present the following article in accordance with the STROBE reporting checklist.

## METHODS

### Study Design

This study aimed to investigate risk factors and prevalence of POAF in isolated off-pump CABG patients. Approval was obtained from the institutional review board of the Ethics Committee of Nanjing University Medical School Affiliated Nanjing Drum Tower Hospital in China. The study was exempted from requiring informed consent from patients as it involved the collection of existing records and/or diagnostic data that were clinically available. Patients were divided into two groups, the new-onset POAF Group (those with postoperative atrial fibrillation) and the non-POAF Group (those with no postoperative atrial fibrillation). The association between the occurrence of new-onset POAF following CABG and other variables was analyzed. The variables analyzed included preoperative, intraoperative and postoperative parameters, outcomes and complications. Risk factors and relevant clinical data were investigated for their relationship with the new-onset POAF.

### Population

Inclusion criteria included adult patients ≥18 years old who underwent isolated elective off-pump CABG surgery without concomitant cardiac or non-cardiac surgical procedures at the Department of Thoracic and Cardiovascular Surgery, the Affiliated Drum Tower Hospital of Nanjing University Medical School between August 2018 and July 2020. Exclusion criteria include patients with a known history of atrial fibrillation (AF), supraventricular arrhythmias, no coronary angiography result, use of anti-arrhythmic drugs other than beta-blockers, digoxin, and calcium-channel blockers.

### Definition and Diagnosis

New-onset POAF was defined as any postoperative AF episode that lasted more than 30 seconds^[8]^ or needed therapy for hemodynamic instability during hospitalization after isolated CABG^[1]^. AF was defined as 1) irregular R-R intervals (when atrioventricular [AV] conduction is present), 2) absence of distinct repeating P waves, and 3) irregular atrial activity^[9]^. Patients were monitored continuously via 12-lead telemetry immediately after surgery and in the intensive care unit (ICU). Standard 12-lead electrocardiography was recorded in the ward until hospital discharge.

Echocardiography examination, cardiac chambers quantification, and function assessment were performed according to the American Society of Echocardiography (ASE) guidelines^[10,11]^ by the echocardiography unit of the Department of Cardiology, the Affiliated Drum Tower Hospital of Nanjing University Medical School.

Linear internal measurements of the left ventricle (LV) and its walls were performed in the parasternal long-axis view. Values were obtained perpendicularly to the LV long axis view and measured at or immediately below the level of the mitral valve leaflet tips. Right ventricular (RV) dimensions are estimated from an RV-focused apical four-chamber view, displaying the largest basal RV diameter, avoiding foreshortening, and reported indexed to body surface area (BSA). Left atrium (LA) anteroposterior (AP) dimension was measured in the parasternal long-axis view using two-dimensional echocardiography (2DE). LA size was measured at the end of the LV systole, when the LA chamber is at its greatest dimension. Right atrium (RA) size is the RA volume obtained in a dedicated apical four-chamber view. LV, LA and RA chamber volumes are measured using 2DE with the biplane disk summation method (modified Simpson’s rule) and reported indexed to BSA.

The sinuses of Valsalva (maximum diameter, usually the midpoint), the sinotubular junction and the proximal ascending aorta were measured at end-diastole, in a strictly perpendicular plane to that of the long axis of the aorta. The leading edge-to-leading edge (L-L) convention was adopted. Values were compared with age- and BSA-related nomograms or to values calculated from specific allometric equations.

A 16-segment model was used to assess regional LV function. A semiquantitative wall motion score was assigned to each segment to calculate the LV wall motion score index as the average of scores of all segments visualized. The scoring system consists of: 1) normal or hyperkinetic, 2) hypokinetic (reduced thickening), 3) akinetic (absent or negligible thickening, *e.g.*, scar), and 4) dyskinetic (systolic thinning or stretching, *e.g.*, aneurysm).

LV diastolic function was measured by four guideline-recommended variables^[12]^ to identify diastolic dysfunction and their abnormal cutoff values are annular e’ velocity: septal e’ <7 cm/s, lateral e’ <10 cm/s, average E/e’ ratio >14, LA volume index >34 mL/m^2^, and peak TR velocity >2.8 m/s. LV diastolic function is normal if more than half of the available variables do not meet the cutoff values to identify abnormal function. LV diastolic dysfunction is present if more than half of the available parameters meet these cutoff values. The study is inconclusive if half of the parameters do not meet the cutoff values.

### Treatment

CABG was performed in all patients, without cardiopulmonary bypass (CPB), by a single surgical team. After anesthesia, a median sternotomy or left anterior lateral incision was performed. The left internal thoracic artery and reverse saphenous vein were adopted for CABG in most instances. Transfusion of red blood cells (RBC), fresh frozen plasma and platelets were recorded. Potassium levels of more than 4 mEq/L were maintained during the whole postoperative period. Routinely medication and management were taken for all patients perioperatively and after initial hospitalization. Briefly, beta-blocker, nitrate, statin, aspirin and ticagrelor were used. Amiodarone was used as first-line therapy for pharmacological conversion to sinus rhythm, while electrical cardioversion was performed in cases of hemodynamic instability. All patients were discharged from the hospital after the surgical incisions were totally healed.

### Statistical Analysis

For all patients, preoperative, intraoperative and postoperative data are systematically and retrospectively collected from electronic medical records systems, paper medical records, nursing care records, and radiology workstations.

Statistical analyses were conducted using SPSS (IBM SPSS Statistics, RRID:SCR_019096) for Windows. Continuous variables are expressed as mean±standard deviation (SD) or median value with quartile value, while the categorical variables are presented as numbers with percentages. Normal distribution was assessed using the Kolmogorov-Smirnov test. The Pearson’s chi-square test was employed to compare the categorical variables, while the unpaired t-test or the Mann-Whitney U test was used to compare the parametric and nonparametric constant variables, respectively.

After univariate associations analysis by logistic regression modeling, all significant variables at a nominal two-tailed *P*≤0.20 were then entered into multivariable logistic models using a combination stepwise selection method. Model entry and retention criteria were set at *P*≤0.20 and *P*≤0.01. Odds ratios (OR) and 95% confidence intervals (CI) were calculated for independent associates of AF. A *P*<0.05 was considered statistically significant.

## RESULTS

### Patients

**Fig. 1:**
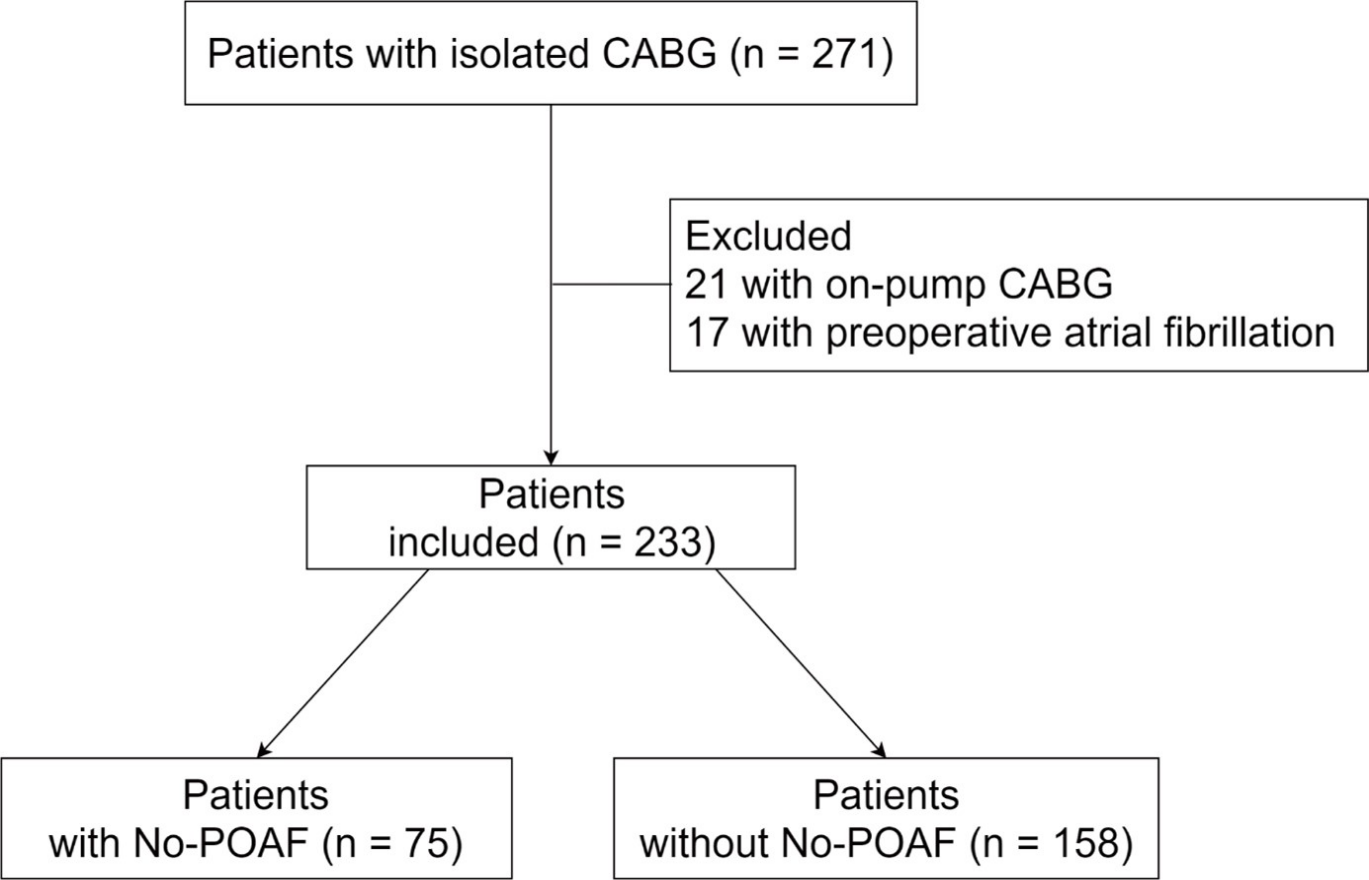
Study profile. CABG=coronary artery bypass grafting; No-POAF=new-onset postoperative atrial fibrillation.

**Fig. 2:**
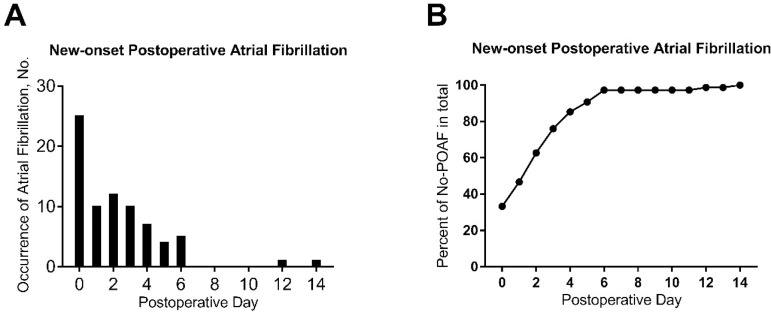
Day of initial occurrence for new-onset postoperative atrial fibrillation. The denominators are 75 patients for new-onset postoperative atrial fibrillation.

Figure 1 summarizes the numbers of screened, included and excluded patients. Of the 271 patients with isolated CABG, 38 were excluded for on-pump CABG or POAF. A total of 233 patients (mean [SD] age, 64.828 [9.997]; 179 men [76.824%]; Table 1) were off-pump CABG patients without POAF. All new-onset POAF patients (75, 32.19%) received an intravenous pump of amiodarone, while 4 of them received electrical cardioversion. New-onset POAF was most common on postoperative day 0 (n=25, 33.33%, Figure 2A) and most of them occurred within 6 postoperative days (n=73, 97.33%, Figure 2B).

**Table 1:** Cohort characteristics.

Parameter	Value*
N	233
Mean age, years (SD)	64.828 (9.997)
Male	179 (76.824%)
BMI, median (IQR)	24.45 (22.185, 26.895)
Mean ejection fraction, % (SD)	52.985% (7.908%)
Diabetes	92 (39.5%)
Hypertension	159 (68.2%)
Preoperative LVEF, median (IQR)	56 (48.75, 59)
New-onset POAF	75 (32.19%)

* Values are n (%) unless indicated otherwise. IQR=interquartile range; LVEF=left ventricular ejection fraction; POAF=Postoperative atrial fibrillation; SD=standard deviation

### In-hospital Variables

The characteristics of both groups are summarized in Table 1. Compared with non-POAF group patients, new-onset POAF patients had a worse baseline condition (advanced age, higher baseline systolic blood pressure, 2-fold non-cardiac surgery history, and 2-fold preoperative diuretic treatment), worse operation condition (2-fold ratio of blood products transfusion), and worse postoperative condition (more drainage in the first 24 hours after surgery, more ICU days, longer total hospital stay, and more patients in postoperative use of dexmedetomidine, digoxin, deslanoside, and norepinephrine). Echocardiography data indicate that new-onset POAF patients had larger preoperative and postoperative left atrium diameter (LAD), and more patients in new-onset POAF had perioperative enlarged cardiac atrium (both left and right).

After univariate associations analysis, 17 variates entered the multivariable logistic regression model (Table 2). The Hosmer-Lemeshow test for the model yielded a score of 7.565 (*P*=0.477). Postoperative left atrial enlargement, postoperative drainage in the first 24 hours and total hospital days were statistically significant, while postoperative (both left and right) atrial enlargement assessed by echocardiography and postoperative use of positive inotropes, including deslanoside, digoxin, norepinephrine and dexmedetomidine, had the largest OR values.

**Table 2:** Multivariable predictors of new-onset POAF.

Predictor	OR (95% CI)	*P*-value
Postoperative right atrial enlargement	7.797 (0.200, 304.294)	0.272
Postoperative left atrial enlargement	3.524 (1.141, 10.886)	0.029
Postoperative use of deslanoside	1.547 (0.783, 3.058)	0.209
Postoperative use of digoxin	1.408 (0.875, 2.265)	0.158
Postoperative use of norepinephrine	1.394 (0.936, 2.076)	0.102
Postoperative use of dexmedetomidine	1.180 (0.814, 1.712)	0.383
Postoperative ICU days	1.110 (0.824, 1.495)	0.494
Hospital length of stay	1.087 (1.010, 1.170)	0.027
Preoperative LAD	1.030 (0.871, 1.218)	0.727
Systolic blood pressure	1.016 (0.991, 1.043)	0.217
Postoperative drainage in the first 24h	1.002 (1.000, 1.004)	0.022
Postoperative BNP	1.000 (1.000, 1.001)	0.249
Age	0.989 (0.935, 1.046)	0.691
Postoperative LAD	0.981 (0.862, 1.117)	0.773
Diuretics	0.832 (0.243, 2.847)	0.769
History of non-cardiac surgery	0.803 (0.216, 2.980)	0.743
Transfusion of blood products	0.608 (0.193, 1.913)	0.395

BNP=brain natriuretic peptide; CI=confidence interval; ICU=intensive care unit; LAD=diameter of left atrial; OR=odds ratio; POAF=postoperative atrial fibrillation

## DISCUSSION

The new-onset POAF is a frequent complication of CABG with unclear mechanisms. Although the heart rate can be easily controlled and AF can be converted to sinus rhythm pharmacologically as well as by electrical cardioversion, new-onset POAF can lead to an increased risk of postoperative complications and long-term negative outcomes. Early work focused on detecting arrhythmias from electrocardiograms, as well as identifying preoperative risk factors from medical records. Advanced age, atrial fibrosis, atrial dilation, hypertension, valvular heart disease, heart failure, obesity, male sex, and CPB have already been reported as risk factors for POAF^[2,7]^. Underlying mechanisms are incompletely defined but include intraoperative and postoperative phenomena, such as inflammation, sympathetic activation and cardiac ischemia, that combine to trigger atrial fibrillation, often in the presence of pre-existing factors, making the atria vulnerable to induction and maintenance of AF^[13]^. Anti-inflammatory properties, such as steroidal anti-inflammatory drugs (corticosteroids) and colchicine, have appeared to be useful in preventing new-onset POAF^[14]^.

In our study, we focused on patients with isolated off-pump CABG to reduce heterogeneity and potential bias. We reviewed 233 patients who received isolated CABG in our center between August 2018 and July 2020. Patients who developed new-onset POAF had a worse condition (advanced age, higher baseline systolic blood pressure, non-cardiac surgery history, more diuretic and positive inotropes treatment, blood product transfusion, 24-hour postoperative drainage, and postoperative intensive care unit days), larger cardiac atrium, and associated with longer hospital stay compared to non-POAF patients.

In our patients, only a few had reduced LVEF and body mass index (BMI) ≥30 kg/m^2^ (World Health Organization criteria for obesity, class I). It seems that there were more diabetes mellitus patients in the new-onset POAF group (34, 45.3%) than in the non-POAF group (58, 36.7%), but differences were not significant (*P*=0.208). New-onset POAF patients had higher systolic blood pressure when admitted to the hospital (new-onset POAF patients [132.05, 20.25], non-POAF group patients [126.22, 18.19], *P*=0.028), even though history of hypertension did not differ between the two groups (new-onset POAF patients [56, 74.7%], non-POAF group patients ]103, 65.2%], *P*=0.147). Significantly more new-onset POAF patients had cardiac atrial enlargement (Table 1). Right atrial enlargement had the largest OR value and there was a huge difference between the two groups (nearly 10-fold; new-onset POAF patients [7, 9.3%], non-POAF group patients [1, 0.6%], *P*=0.003). Eight patients had right atrial enlargement among all 233 patients, 7 (87.5%) of whom developed new-onset POAF. More new-onset POAF patients received positive inotropes treatment both preoperatively and postoperatively. Postoperative use of dexmedetomidine, digoxin, deslanoside and norepinephrine were significantly different. Multivariable logistic regression results indicated that atrial dilation, use of positive inotropes, higher baseline systolic blood pressure, and postoperative drainage were risk factors. The 24-hour drainage was also reported as a risk factor for postoperative AF in another study^[15]^. Right atrial dilation had an OR value of 7.797 (95% CI, 0.200, 304.294), and left atrial dilation had an OR value of 3.524 (1.141, 10.886), thus may provide the potential of echocardiography as a useful tool for new-onset POAF prediction.

Norepinephrine has also been shown a risk factor for AF in other studies. In 2017, a prospective, randomized, double-blind trial revealed a lower incidence of AF in the vasopressin group *versus* norepinephrine group in patients after cardiac surgery. This may be due to increased adrenergic tone through beta-1 receptors, resulting in an increased atrial ectopic activity and, consequently, AF^[16]^.

Several studies have suggested dexmedetomidine as a potential strategy for AF prevention^[17]^. Possible mechanisms of dexmedetomidine are 1) reducing myocardial ischemia-reperfusion injury and improving myocardial perfusion^[18]^, 2) inhibiting the inflammatory response induced by cardiac surgery and CPB^[18]^, 3) providing a lower adrenergic tone^[19,20]^, 4) leading to alterations in calcium currents across the cardiomyocyte cell membrane, resulting in an increased effective refractory period^[21]^. The opposite result of our data could be explained as a worse condition and heart dysfunction compared to non-AF patients since dexmedetomidine was used as a positive inotrope in our study.

The most accepted mechanisms underlying AF are re-entry and ectopic activity^[22]^. Re-entry means the continued impulse propagation around a functional or structural obstacle. The occurrence of re-entry requires a vulnerable substrate and ectopic electrical activity works as a trigger of re-entry. AF is associated with atrial fibrosis and electrical remodeling, resulting in re-entry-promoting shortening of atrial repolarization. Otherwise, cardiac surgery triggers inflammation in the heart and makes it susceptible to the incidence of AF^[14]^.

Conventional management strategies for POAF include preventing thromboembolic events, controlling the ventricular rate response and restoring or maintaining sinus rhythm while there is no clear benefit between rate- or rhythm-control therapy^[17]^ Early initiation of oral anticoagulation was not associated with reduced long‐term risk of ischemic stroke or thromboembolism, but with increased risk of major bleeding^[23]^. Patients presenting for cardiac surgery often receive beta-blockers as a part of their chronic medical therapy and the continuation of beta-blockers in the perioperative period carries a class I recommendation^[24]^. However, nearly 1/3 of patients developed new-onset POAF, even though all patients in our study obeyed the guideline recommendation, thus there is an urgent need for a better prevention method.

In this study, a series of factors differ significantly between new-onset POAF patients and non-POAF patients after isolated CABG. These factors are similar to previous literature and are rational, since basal conditions such as uncontrolled hypertension and heart failure (need for diuretics and positive inotropes) resulted in atrial remodeling and dilation, thus increasing susceptibility to AF, while negative stimulation (inflammation caused by cardiac surgery and/or blood products transfusion^[25]^, increased adrenergic tone caused by medical treatment) triggered the occurrence of AF. Accordingly, we propose a comprehensive prevention model that consists of hypertension control, atrial remodeling retention/restoration, and anti-inflammation besides conventionally established prophylaxis (beta-blocker, amiodarone and sotalol).

### Limitations of the Study

Limitations of our study include its single-center retrospective observational design and its limited sample size. Second, the lack of data for some variables may produce bias in the result. For example, 43 patients had no preoperative data on LAD, among which 15 patients developed new-onset POAF. However, 10 of the 15 new-onset POAF patients had a postoperative LAD ≥40 mm. We run a linear regression for preoperative and postoperative LAD, finding a significant linear relationship between these two variables (*P* <0.001). Taken together, the absence of preoperative LAD in 43 patients did not disturb the conclusion that preoperative LAD could be a predictor of new-onset POAF. Third, no systematic electrolyte concentrations were obtained. Thus, the potential impact of systematic electrolyte concentrations on new-onset POAF could not be discovered. At last, we focused on off-pump CABG patients to reduce heterogeneity and potential bias, thus excluding some variates such as CPB and special anti-arrhythmic drugs. This will limit the generalization of this study.

## CONCLUSION

In summary, this single-center retrospective observational study focuses on new-onset POAF in isolated off-pump CABG patients. Our data depicts a map for this type of patient, highlighting the advantage of echocardiography as a useful tool for predicting new-onset POAF. Patients with atrial enlargement are highly predictive of POAF. Careful monitoring and timely intervention should be considered for these patients.

This study also proposes that uncontrolled hypertension and heart failure may lead to atrial remodeling and dilation, providing susceptibility to AF, while negative operation-related stimulation may produce inflammation, leading to ectopic electrical activity. Thus, future prevention strategies may focus on hypertension control, atrial remodeling retention/restoration, and anti-inflammation.

### Data Availability

Researchers who want to re-analyze the data can get primary patient characteristics data from the corresponding author for data analysis, manuscript writing, and publication such as meta-analysis or systematic review after publication of this article.
